# Multidisciplinary Outpatient Cancer Rehabilitation Can Improve Cancer Patients’ Physical and Psychosocial Status—a Systematic Review

**DOI:** 10.1007/s11912-020-00979-8

**Published:** 2020-10-01

**Authors:** Daisy Kudre, Zhehui Chen, Aline Richard, Sophie Cabaset, Anna Dehler, Margareta Schmid, Sabine Rohrmann

**Affiliations:** Division of Chronic Disease Epidemiology; Epidemiology, Biostatistics and Prevention Institute, University of Zurich, Hirschengraben 82, CH-8001 Zurich, Switzerland

**Keywords:** Cancer rehabilitation, Outpatient rehabilitation, Multidisciplinary rehabilitation, Systematic review

## Abstract

**Purpose of Review:**

This systematic review aimed to determine the effects of interdisciplinary/multidisciplinary outpatient rehabilitation programmes by looking at physical, psychosocial and return to work status of adult cancer patients.

**Recent Findings:**

There is growing evidence that emphasizes the importance of interdisciplinary/multidisciplinary rehabilitation especially in outpatient care, which addresses the complex and individual needs of cancer patients. Many studies focus on measuring the effect of individual rehabilitation interventions.

**Summary:**

Randomized controlled trials (RCTs) and before-after studies examining the effects of interdisciplinary/multidisciplinary outpatient rehabilitation programmes were included in this systematic review. The electronic literature search was conducted in MEDLINE, EMBASE, CINAHL, Cochrane Central Register of Controlled Trials and PEDro. The PICO statement was used for selection of the studies. Six randomized controlled trials and six before-after studies were included. Interdisciplinary/multidisciplinary outpatient cancer rehabilitation programmes improved physical and/or psychosocial status of cancer patients. However, non-significant changes in a variety of single physical and psychosocial measures were also common.

The findings of the systematic review indicate that interdisciplinary/multidisciplinary outpatient cancer rehabilitation can improve cancer patients’ physical and psychosocial status. This review is limited by the narrative approach due to the heterogeneity of outcome measures. To evaluate effects of rehabilitation, better comparable studies are necessary. Further research is needed in regard to long-term outcomes, effects on return to work status and on the associations depending on cancer type.

## Introduction

The number of people living with a cancer diagnosis is constantly increasing due to an aging population and successful cancer treatment. Thus, cancer is progressively seen as a chronic disease [1]. However, cancer itself and its treatment can result in a wide range of physical and psychological impairments, e.g. pain, fatigue, cognitive difficulties, anxiety and depression, having a negative effect on cancer patients’ quality of life (QoL) [2].

Cancer rehabilitation has been proven effective in decreasing the side effects of cancer and cancer treatment. WHO has defined rehabilitation as a “set of interventions designed to optimize functioning and reduce disability in individuals with health conditions in interaction with their environment” [3]. Studies mostly focus on measuring the effect of individual rehabilitation interventions after cancer diagnosis or treatment, such as physical activity [4]. For example, it was observed in a meta-analysis that physical activity interventions helped reduce cancer-related fatigue and anxiety and increased the functional QoL as well as aerobic fitness and muscle strength [5]. Psychological interventions also reduced fatigue [6] and anxiety [7]. Interventions such as consultation with an occupational physician supported cancer survivors in returning to the workplace [8].

However, growing evidence emphasizes the importance of interdisciplinary and multidisciplinary rehabilitation, which addresses the complex needs of cancer patients through a more comprehensive approach compared with monodisciplinary care [9–11]. Interdisciplinary rehabilitation is defined as a programme where several health care specialists agree on mutual goals while working on these goals in individual sessions. Regular meetings and coordinated information flow are an integral part of such programmes. Multidisciplinary rehabilitation, in contrast, does not necessarily include synergic teamwork [12].

To the best of our knowledge, two systematic reviews have been conducted on the effects of inpatient and outpatient cancer rehabilitation, but they observed mixed effects [13, 14]. Scott et al. examined the effects of multidimensional, i.e. often monotherapy that focused on at least two interventional aspects (e.g. counselling to foster physical activity and better stress management), but not necessarily multidisciplinary interventions [14•]. Mewes et al. focused also on multidimensional intervention and included also multidisciplinary interventions [13•]. Thus, there is still a lack of knowledge concerning rehabilitation that is truly multidisciplinary, not only multidimensional. Furthermore, little is known about the effects on physical and psychosocial health particularly for outpatient cancer rehabilitation, which refers to rehabilitation that is offered at a hospital or medical facility without being admitted. Such outpatient programmes can be longer than inpatient programmes, but are usually less intensive.

The aim of the study was to review the characteristics of interdisciplinary/multidisciplinary outpatient rehabilitation programmes in research published so far and to assess the effects of rehabilitation programmes for physical, psychosocial and/or return to work status of adult cancer patients. Both interdisciplinary rehabilitation and multidisciplinary rehabilitation were included and are referred to as ‘multidisciplinary’ in this study. To the best of our knowledge, this is the first systematic review to assess the effects of multidisciplinary outpatient cancer rehabilitation (MOCR). Studies with an inpatient or a multidimensional setting were not included.

## Material and Methods

### Search Strategies

Electronic databases for the literature search included MEDLINE, EMBASE, CINAHL, Cochrane Central Register of Controlled Trials and PEDro. The search was performed on June 19, 2018. Search terms focused on cancer and interdisciplinary or multidisciplinary rehabilitation and were used in combinations and adaptions for each electronic database, according to the expertise of a librarian. Randomized controlled trials (RCTs), as well as quantitative study types, were included due to little available research in the field of MOCR, thus providing the potential to supplement RCT evidence.

### Study Selection

The PICO statement was used to set criteria for considering studies for this review.

Only studies with adult cancer patients (≥ 18 years old), with any cancer type and stage, were included (Population). Studies with multidisciplinary/interdisciplinary outpatient cancer rehabilitation, which were defined as conducting two or more Interventions, were included if they have been delivered during or up to 2 years after the end of the primary treatment. Controls were not specified. Outcomes assessed the physical and/or psychosocial effectiveness and/or the return to work status.

Two reviewers independently assessed titles and abstracts of the retrieved articles, categorizing them into ‘inclusion criteria fulfilled [A]’, ‘inclusion criteria not fulfilled [B]’ or ‘unclear [C]’. Inclusion criteria consisted of four aspects: diagnosis of cancer, rehabilitation, study design and cancer patients being adult. The decisions of the two reviewers were compared and articles rated as [A-A] or [A-C] were included for full-text screening. For combinations of [A-B], further evaluations followed to decide whether to include the article or not. Then, two reviewers independently evaluated full texts of all potentially eligible papers. Briefly, the inclusion criteria of the full-text screening consisted of the minimum of two different interventions of which one, but not both, included physical training. Further inclusion criteria were the timeframe of rehabilitation in an outpatient setting (during or up to 2 years after the end of cancer treatment), the quantitative study design (RCTs or before-after studies) and the use of at least one assessment tool before and during or at the end of the rehabilitation. Additionally, disagreements and unclear decisions were resolved by consensus.

### Data Extraction and Risk of Bias

The data was independently extracted from the two review authors using a predeveloped data extraction template which covered general aspects (e.g. authors, title), methods, participants, interventions and outcome.

The Cochrane Collaboration’s risk of bias tool [15] was used to assess the risk of bias resulting from random sequence generation (selection bias), allocation concealment (selection bias), blinding of participants and personnel (performance bias), blinding of outcome assessment (detection bias), incomplete outcome data (attrition bias), selective reporting (reporting bias) and bias due to confounding in each study. If no conclusion about a bias could be drawn due to the nature of the study (e.g. blinding of participants in either receiving or not receiving rehabilitation interventions), or the study design (e.g. random sequence generation in non-randomized trials), the entry was judged with ‘high risk of bias’. Bias due to confounding was added to the original Cochrane tool of bias list to evaluate further bias in non-randomized studies.

This systematic review was registered in PROSPERO (CRD42018100145).

## Results

### Study Selection

**Fig. 1:**
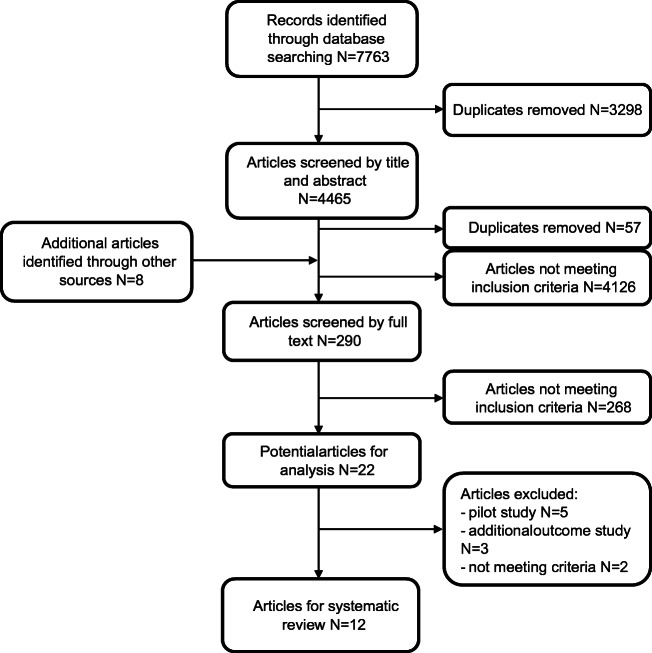
Workflow of the systematic review

Database searching resulted in 7763 articles, and after removing duplicates, 4465 articles were screened by title and abstract. Two hundred ninety articles were considered for full-text screening. Finally, 12 articles were included for the systematic review (Fig. 1).

### Study Characteristics

Five of the studies included were carried out in Denmark [16–19], two in the USA [20, 21], and one each in South Korea [22], Australia [23], Belgium [24], the Netherlands [25] and Norway [26]. Out of 12 studies included, six were randomized controlled trials [16–18, 20–22], two were controlled before-after studies [23, 24] and four were uncontrolled before-after studies [19, 25–27]. In the following text, RCTs and before-after studies were analyzed separately due to the differences in methodological quality and level of evidence.

#### Randomized Controlled Trials

Detailed information is listed in Table 1. In summary, the sample size of the six RCTs varied from 65 [22] to 269 cancer patients [16] resulting in a total of 862 participants. Four of the RCTs [16, 18, 20, 21] included participants with different types of cancer.

**Table 1:** Characteristics of the participants and studies selected in randomized controlled trials and before-after studies

Author, year of publication	Country	Participants		Multidisciplinary Interventions					Control group	Outcome	
		Sample size*	Cancer site	Timing of rehabilitation	Interventions	Intensity	Duration of the programme	Professionals		Measures	Time point of assessment
Randomized controlled studies											
Adamsen, 2009	Denmark	269 I 135, C 134	21 different types (17 solid tumors, 4 malignant hematological diseases)	During treatment	High-intensity physical training, relaxation training, body awareness training, massage	9 h per week (physical training 3 times per week for 90 min, relaxation 3 times per week for 30 min, massage 2 times a week for 30 min, body awareness training once per week for 90 min)	6 weeks	Physiotherapists, specially trained nurse	Standard medical care	EORTC QLQ-C30, MOS SF-36, VO2max, 1RM, physical activity questionnaire	Baseline, 6 weeks after baseline (post-rehabilitation)
Cho, 2006	South Korea	65 I 34, C 31	Breast cancer	After primary treatment	Psychology-based education, physical training, peer support group activity	5.5 h per week (education once per week for 90 min, exercise twice per week for 90 min, group activity once per week for 60 min)	10 weeks	Psychology-based education: oncology nurse, surgeon, dietician, and image consultant. Physical training: not specified. Peer support group activity: researcher	No rehabilitation	Range of motion of the affected shoulder joint, psychosocial adjustment (18 items, 4-point scale)	Baseline, 10 weeks after baseline (post-rehabilitation)
Clark, 2013	USA	129 I 65; C 64	Different types (brain, head and neck, lung, gastrointestinal, other)	During treatment	Physical therapy, cognitive behavioural therapy, education around cancer management, relaxation, spirituality training, social therapy, phone counselling	4.5 h per week (3 sessions per week, 90 min each)	2–4 weeks + phone counselling for 22 weeks	Physical therapist, clinical psychologist/ psychiatrist, advanced practiced nurse, hospital chaplain, clinical social worker	Standard medical care	FACT-G (The Caregiver Quality of Life Index-Cancer Scale—not of our interest)	Baseline, 4 weeks after baseline (post intensive rehabilitation), 27 weeks after baseline (post less intensive rehabilitation)
Jarden, 2016	Denmark	70 I 34, C 36	Acute leukemia	During treatment	Physical training (including relaxation), health counselling sessions	Ca 3 h per week (physical training 3 times per week 60 min) + 30–60-min health counselling at W1, W6, W12	12 weeks	Not specified	Standard medical care	6MWD, VO2max, FACT-An, HADS, EORTC QLQ-C30, sit to stand, biceps curl, physical activity questionnaire, MOS SF-36	Baseline, 6 weeks after baseline (mid-rehabilitation), 12 weeks after baseline (post-rehabilitation)
Midtgaard, 2013	Denmark	214 I 106, C 108	Different types (60% breast cancer + bowel, ovaries, uterus, testes, hematological malignancies, other)	After treatment	Physical training, counselling sessions	Physical training: 1 session per week, 90 min each; counselling sessions: 9 sessions per year, 1–2 h each	12 months	Trained psychologist (counselling); not specified (physical training)	Health evaluation (3 sessions per year, 15 min each; education on the health benefits of regular exercise)	Saltin and Grimby questionnaire, incremental exercise test, 1RM, HRQOL, EORTC QLQ-C30, HADS, MOS SF-36, VO2max	Baseline, 6 months after baseline (mid-rehabilitation), 12 months after baseline (post-rehabilitation).
Rummans, 2006	USA	115 I 57, C 58	Different types (brain, head and neck, lung, ovarian, gastrointestinal, other)	During treatment	Physical therapy, cognitive behavioural therapy, social therapy, emotional support intervention, spiritual intervention	3–4.5 h per week (8 sessions over 3 weeks, 90 min each)	3–4 weeks	Physical therapist, psychiatrist/psychologist, advanced practice nurse, hospital chaplain, social worker	Standard medical care	Spitzer QOL Uniscale and LASAs of QOL; Symptom Distress Scale, POMS Short Form; FACIT-Spiritual wellbeing	Baseline, 4 weeks after baseline (post-rehabilitation), 8 weeks after baseline (post-rehabilitation), 27 weeks after baseline (post-rehabilitation)
Before-after studies											
Andersen, 2006	Denmark	88	Different types (45 solid tumors, 9 malignant hematological diseases)	During treatment (chemotherapy)	Physical training, relaxation, massage, body awareness training	9 h per week	6 weeks	Trained physiotherapists, specially trained nurse	No comparison group	Common Toxicity Criteria—CTC questionnaire (symptoms and side effects)	Daily self-assessment from baseline to 6 weeks after baseline (post-rehabilitation)
Gordon, 2005	Australia	275 I 31, C1 36; C2 208	Breast cancer	After treatment	Physical training targeting shoulder movement, education, psychosocial advice, peer support	1–2 h per week	8 weeks	Exercise physiologist	C1: home-based physiotherapy intervention group; C2: no rehabilitation	FACT-B, DASH	Baseline, 8 weeks after baseline (post-rehabilitation), 6 months after the diagnosis (post-rehabilitation), 12 months after the diagnosis (post-rehabilitation)
Leclerc, 2018	Belgium	209 I 103; C 106	Breast cancer	After primary treatment	Physical training, psychoeducational sessions	6.5 h per week (4.5-h physical training, 2-h psychoeducation)	12 weeks	Physiotherapist, psychologist, professor in physiotherapy and rehabilitation, dietician, neurologist	C: no rehabilitation	EORTC QLQ-C30, EQ-5D, FACIT-Fatigue, STAI, HADS, FPACQ	Baseline, 3 months after baseline (post-rehabilitation), 6 months after baseline (post-rehabilitation), 12 months after baseline (post-rehabilitation), 24 months after baseline (post-rehabilitation)
Leensen, 2017	Netherlands	95	Different types (breast [84%], colorectal, non-Hodgkin’s lymphoma, other).	During treatment (chemotherapy)	Physical training, personal occupational counselling	2 h per week for physical exercise +1–3 counselling sessions per 12 weeks	12 weeks	Physiotherapist, oncological occupational physician	No comparison group	Work resumption questionnaire, VAS, self-efficacy scale, WAI, WLQ, 1-RM, VO2 peak test, MFI, SQUASH, EORTC-QLQ-C30	Baseline, 6 months after baseline (post-rehabilitation), 12 months after baseline (post-rehabilitation), 18 months after baseline (post-rehabilitation)
Seibaek, 2016	Denmark	371,217 cancer patients 154 relatives	Gynecological cancer (ovarian, endometrial, cervical, vulva, other gynecological)	After treatment	Information, physical training, and supportive group sessions	3 h per day once a week	4 weeks	Nurse specialists, chief surgeon, physiotherapist, body therapist, sexologist, psychotherapist	No comparison group	SF-36	Baseline, 12 months after baseline (post-rehabilitation)
Thorsen, 2016	Norway	115	Different types (breast, gynecological, lymphoma, esophagus)	After primary treatment	Physical training, patient education, group discussion	4–5 h per day once a week	7 weeks	Social worker, health practitioner, physiotherapist	No comparison group	Self-reported work status, EORTC QLQ-C30, Fatigue Questionnaire	Baseline, 6 months after baseline (post-rehabilitation)

*I* intervention group, *C* control group

In four RCTs, MOCR was provided for cancer patients still undergoing chemotherapy and/or radiation therapy [16, 17, 20, 21]. MOCR programmes all included physical training and some type of psychological counselling interventions, partly also relaxation methods. They lasted from 2 to 4 weeks [21] to 12 months [18], and the intensity varied from 1.5 h per week [18] to 9 h per week [16]. Cancer patients allocated to the control group received standard medical care only, except for one study, in which a less intense intervention was applied [18].

#### Before-After Studies

The sample size of studies included varied from 88 [27] to 275 cancer patients [23], and the total sample size for the six before-after studies was 1153 participants. Three studies [25–27] included participants with different types of cancer, two studies [23, 24] included breast cancer patients and one study [19] patients diagnosed with a gynecological cancer.

In two studies [25, 27], the rehabilitation programme included cancer patients undergoing chemotherapy; in the other 4 studies [19, 23, 24, 26], rehabilitation was provided after completion of (primary) treatment. MOCR programmes in before-after studies included physical training as well as some type of psychological counselling. They lasted 4 [19] to 12 weeks [24, 25] with an intensity ranging from 1 to 2 h per week [23] to 9 h per week [27]. One study included two control groups receiving either home-based physiotherapy interventions or no rehabilitation [23] and the other before-after study with a control group applied no rehabilitation to the controls (Table 1) [24].

### Outcome Assessment

The most commonly used outcome measurements among the six included RCTs were the QoL of cancer patient’s questionnaire (EORTC QLQ-C30) and the Medical Outcomes Study 36-Item Short Form Survey Instrument (MOS SF-36), both used in three RCTs [16–18]. In addition, three RCTs [17, 20, 21] used one or more subscales of Functional Assessment of Chronic Illness Therapy (FACIT); another three [16–18] measured cardiorespiratory fitness via VO2max/peak test. Others used less known QoL questionnaires or psychosocial adjustment scales [22]. Similarly, in before-after studies, the most commonly used outcome measure was EORTC QLQ-C30 in three [24–26], a subscale of FACIT in two [23, 24] and return to work status in another two studies [25, 26] (Table 1).

**Fig. 2:**
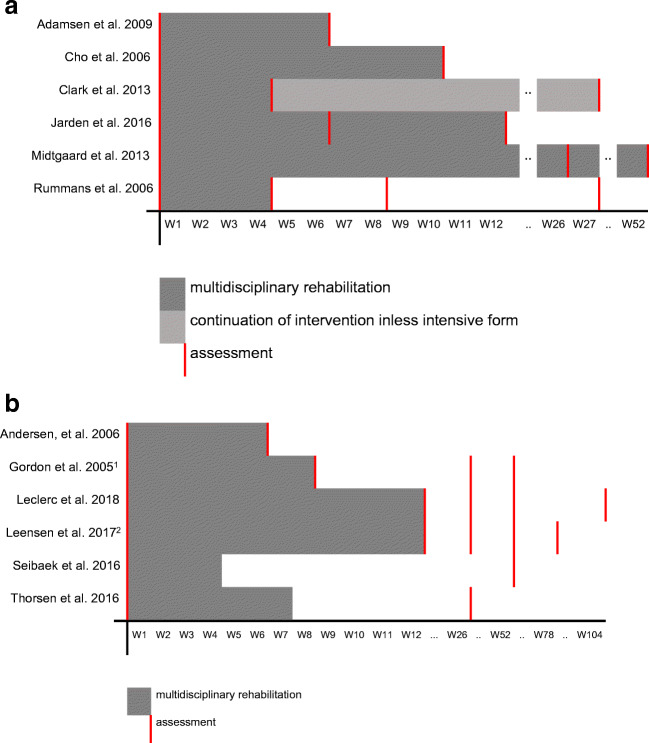
**a** Duration of multidisciplinary rehabilitation and time point of outcome assessment in RCTs. **b** Duration of multidisciplinary rehabilitation and time point of outcome assessment in before-after studies

Outcomes were assessed at various time points. In all RCTs, assessments were done at baseline and directly after the end of the rehabilitation (Fig. 2a). Only one study [21] assessed the outcome of rehabilitation not only after the intervention, but also approximately 4 and 23 weeks after the end of the intervention. In before-after studies, three studies [23, 24] assessed outcomes at baseline and directly after the end of the rehabilitation. Except for one study [27], all before-after studies measured outcomes also some time after the end of rehabilitation, between 6 weeks and 24 months after baseline (Fig. 2.b).

### Risk of Bias

**Fig. 3:**
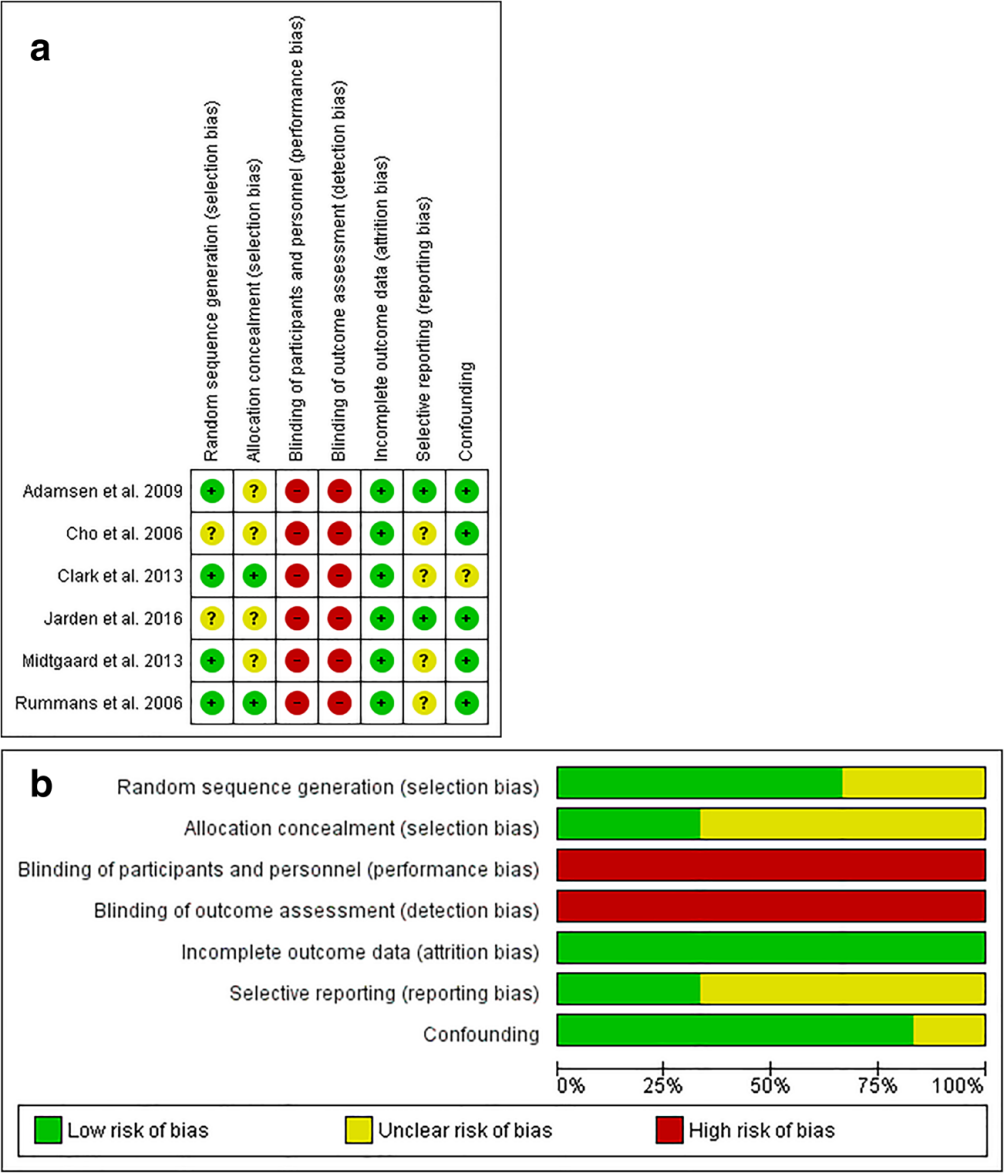
**a** Assessment of ‘risk of bias’ for the included RCTs (*N* = 6). **b ‘**Risk of bias’ graph for RCTs: judgments about each risk of bias item presented as percentages across all included studies (*N* = 6)

Two RCTs [17, 22] did not clearly mention the method of random sequence generation and were classified as being of unclear risk of bias. Four RCTs [16–18, 22] did not clearly describe the method of allocation concealment and thus were classified as being of unclear risk of bias. All RCTs were classified with a high risk of performance bias as it was not possible to blind the participants and personnel to the allocated interventions. Also, outcome assessments included patient self-reports, which were not blinded. Therefore, all studies were classified as being at a high risk of detection bias. All studies reported the numbers and reasons for missing data, and either stated that the drop-outs were equal in intervention and control group and/or performed an appropriate analysis to prove that the missing data had no impact on the results. Hence, all studies were classified as being at low risk of attrition bias. Study protocols of two RCTs were available [16, 17], and as all the prespecified primary and secondary outcomes have been reported in the publications, both studies were classified with low risk of reporting bias. However, for the rest of the RCTs, there was insufficient information to allow for any judgment other than ‘unclear risk of bias’. Bias due to confounding was classified as being low in all RCTs except for the study of Clark et al. ( [20)], which was classified as unclear (Fig. 3 a and b).

**Fig. 4:**
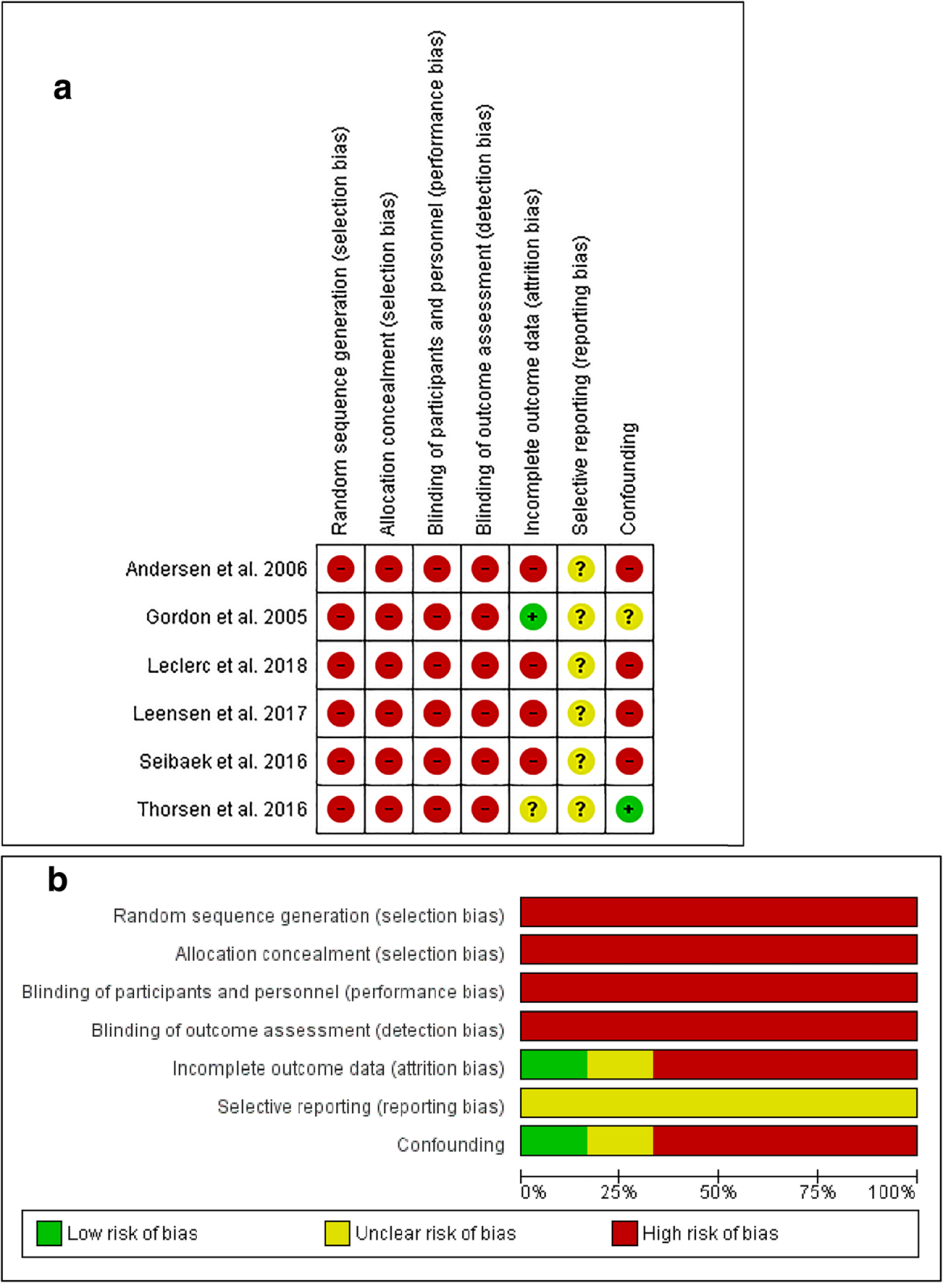
**a** Assessment of ‘risk of bias’ for the included before-after studies (*N* = 6). **b ‘**Risk of bias’ graph for before-after studies: judgments about each risk of bias item presented as percentages across all included studies (*N* = 6)

Overall, all before-after studies were assessed as being of high risk of bias in most categories. This is due to the study design and the nature of the interventions. Two before-after studies [23, 24] were controlled studies, which neither used randomization nor allocation concealment and were, therefore, rated as being of high risk of selection bias. The remaining before-after studies did not have a control group, which indicates a high risk for selection bias. As a result of the nature of the interventions and the study design in all before-after studies, it was not possible to blind the participants. Due to a high drop-out rate (around 30%), four studies [19, 24, 25, 27] were assessed as being of high risk of attrition bias. Study protocols of before-after studies could not be detected, and thus were all classified as being of unclear risk of reporting bias. Four studies [19, 24, 25, 27] did not control for confounding and were, therefore, classified as being of high risk of confounding bias (Fig. 4 a and b).

### Results of Individual Studies

The significant and non-significant results of the measured outcomes of all RCTs and before-after studies are listed in Table 2. Regarding the physical status (i.e. objectively measured physical components and/or physical well-being) of cancer patients, each of the six RCTs observed statistically significant improvements in at least one of the outcomes measured. The most commonly reported significantly improved outcome was physical capacity/cardiorespiratory fitness, measured by VO2 max/peak exercise test [16–18]. Before-after studies came to similar conclusions, although fewer studies measured physical strength, but rather focused on self-reported physical well-being. Four studies observed statistically significant improvements in the physical status of cancer patients [19, 24–26]. Regarding the psychosocial status of cancer patients, five out of six RCTs observed statistically significant improvements of at least one outcome measure [17, 18, 20–22]. Four before-after studies showed that patients who underwent MOCR had improved their psychosocial status in a long-term view [19, 24–26]. Two before-after studies [25, 26] were designed primarily to enhance return to work of cancer patients. Six months after the start of rehabilitation, 59% and 64% of cancer patients, respectively, had returned to work and in one study 83% of cancer patients had returned to work 18 months after the start of rehabilitation [25]. Improvements in the physical and/or psychosocial status of cancer patients have been detected for rehabilitation programmes that took place during the cancer treatment or up to 2 years after the cancer treatment. Improvements were also observed in patients with site-specific diagnoses and in cancer patients with different types of cancer.

**Table 2:** Overview of results of individual studies in randomized controlled trials and before-after studies

Author, year of publication	Time point of assessment	Results of the intervention group compared with control group	
		Significant results, i.e. health improvements	Non-significant results
Randomized controlled trials			
Adamsen, 2009	Week 6 after baseline (post-rehabilitation)	•Cardiorespiratory fitness (VO2max) •Muscle strength (1RM) EORTC QLQ-C30: •Less Fatigue MOS SF-36: •Physical functioning •Role physical •Role emotional •Mental health •Vitality •Mental component scale (summary scale) •Physical component scale (summary scale)	•Self-reported physical activity EORTC QLQ-C30: •QoL •Role functioning •Physical functioning •Emotional functioning •Cognitive functioning •Social functioning •Nausea and vomiting •Pain •Dyspnoea •Insomnia •Appetite loss •Constipation •Diarrhoea •Financial difficulties MOS SF-36: •Bodily pain •General health perceptions •Social functioning
Cho, 2006	Week 10 after baseline (post-rehabilitation)	•Psychosocial adjustment •Quality of life Range of motion of the affected shoulder joint: •Extension •Abduction •External rotation •Internal rotation •Total score	Range of motion of the affected shoulder joint: •Flexion
Clark, 2013	Week 4 after baseline (post intensive rehabilitation)	FACT-G scales: •Quality of life •Physical well-being •Functional well-being	FACT-G scales: •Social well-being •Emotional well-being
	Week 27 after baseline (post less-intensive rehabilitation)		FACT-G scales: •Quality of life •Physical well-being •Functional well-being •Social well-being •Emotional well-being
Jarden, 2016	Week 6 and week 12 after baseline (post-rehabilitation)	•Physical function (6MWD). •Cardiovasculatory fitness (VO2max) •Muscle strength (Left biceps curl, Right biceps curl) •Self-reported leisure-time physical activity •Physical well-being (FACT-An) •Functional well-being: (FACT-An) •Emotional well-being: (FACT-An) •Fatigue (FACT-G) •Total score FACT-G •Trial outcome Index •Total FACT-An •Physical health (SF36) •Anxiety (HADS) •Depression (HADS) EORTC QOL-C30: •Nausea and vomiting •Global Health •Emotional functioning	•Social well-being (FACT-An) •Fatigue (EORTC QOL-C30) Results that were not described in the article, but probably are not significant: •Subscale of FACT-An •Subscales of FACT-G •Subscales of EORTC QOL-C30
	6 months after baseline (post-rehabilitation)	•Cardiorespiratory fitness (VO2peak absolute, VO_2peak_ relative, peak power output, time to exhaustion) •Upper and lower muscular strength (1RM) •Cognitive functioning (EORTC QLQ-C30)	•Cardiorespiratory fitness (HR67watt) EORTC QLQ-C30: •QoL •Physical functioning •Role functioning •Emotional functioning •Social functioning •Fatigue •Nausea and vomiting •Pain •Dyspnoea •Insomnia •Appetite loss •Constipation •Diarrhea •Financial difficulties •Depression (HADS) •Anxiety (HADS) SF-36: •Physical functioning •Role physical •Bodily pain •General health perceptions •Vitality •Social functioning •Role emotional •Mental health •Mental component scale (summary scale) •Physical component scale (summary scale)
Midtgaard, 2013	12 months after baseline (post-rehabilitation)	•Self-reported physical activity level •Cardiorespiratory fitness (VO2peak absolute, peak power output, time to exhaustion) •Upper and lower muscular strength (1RM) •Depression (HADS) •Mental health (SF-36)	•Cardiorespiratory fitness (VO_2peak_ relative, HRpeak, HR67watt) •Anxiety (HADS) EORTC QLQ-C30: •QoL •Physical functioning •Role functioning •Emotional functioning •Cognitive functioning •Social functioning •Fatigue •Nausea and vomiting •Pain •Dyspnoea •Insomnia •Appetite loss •Constipation •Diarrhoea •Financial difficulties SF-36: •PF •Physical functioning •Role physical •Bodily pain •General health perceptions •Vitality •Social functioning •Role emotional •Mental health •Mental component scale (summary scale) •Physical component scale (summary scale)
Rummans, 2006	Week 4 after baseline (post-rehabilitation)	LASA: •Overall quality of life •Overall spiritual well-being POMS: Lower emotional distress: •Tension/anxiety •Confusion/bewilderment	LASA: •Cognitive •Physical •Emotional •Social •Pain frequency •Pain severity •Fatigue •Social support •Financial •Legal POMS: •Total score Symptom distress scale: •Physical symptoms Functional Assessment of Chronic Illness Therapy scale: •Spiritual well-being
	Week 8 after baseline (post-rehabilitation)	Same measures, no significant results	
	Week 27 after baseline (post-rehabilitation)	Same measures, no significant results	
Before-after studies			
Andersen, 2006	Daily self-assessment from baseline until 6 weeks after baseline (post-rehabilitation)	•Myalgia •Other pain •Total pain •Symptoms/side effects	•Lack of appetite •Nausea •Vomiting •Diarrhoea •Paraesthesia •Constipation •Physical fatigue •Mental fatigue •Treatment-related fatigue, •Arthralgia
Gordon, 2005	8 weeks after baseline (post-rehabilitation)	DAART clinically but not statistically significant: •Functional well-being, •Arm function •Global HRQoL •Upper-body function STRETCH: •FACT-G •FACT-B •FACT-B+4	DAART: •Physical well-being •Functional well-being •Breast cancer •Arm morbidity •FACT-G •FACT-B •FACT-B+4 •DASH STRETCH: •Physical well-being •Functional well-being •Breast cancer •Arm morbidity •DASH
	6 to 12 months after baseline (post-rehabilitation)	Two intervention groups (early home based physiotherapy DAART and group-based exercise and psychosocial intervention STRETCH) compared with a control group and across time	
		Differences across time: •Physical well-being •Breast cancer •FACT-G	Differences across time: •Functional well-being •Arm Morbidity •FACT-B •FACT-B+4 •DASH Differences across interventions: •Physical well-being •Functional well-being •Breast cancer •Arm morbidity •FACT-G •FACT-B •FACT-B+4 •DASH
Leclerc, 2018	3, 6, 12 and 24 months after baseline (post-rehabilitation)	Differences between experimental and control group:	
		EORTC QLQ-C30: •Physical functioning •Role functioning •Emotional functioning •Cognitive functioning •Social functioning •Fatigue •Dyspnoea •Financial difficulties •QoL (EQ-5D)	EORTC QLQ-C30: •QoL •Nausea and vomiting •Pain •Insomnia •Appetite loss •Constipation •Diarrhoea •Fatigue (FACIT) •Anxiety state (STAI) •Level of physical activity (FBACQ)
		Differences across time:	
		EORTC QLQ-C30: •QoL •Physical functioning •Role functioning •Emotional functioning •Cognitive functioning •Social functioning •Fatigue •Pain •Dyspnea •Insomnia •Appetite loss •Constipation •Financial difficulties •QoL (EQ-5D) •Fatigue (FACIT) •Anxiety state (STAI) •Anxiety trait (STAI) •Level of physical activity (FBACQ)	EORTC QLQ-C30: •Nausea and vomiting •Diarrhoea
		Interaction of group and time:	
		EORTC QLQ-C30: •QoL •Role functioning •Physical functioning •Emotional functioning •Fatigue •Pain •Insomnia •Diarrhea •QoL (EQ-5D) •Fatigue (FACIT) •Anxiety state (STAI) •Anxiety trate (STAI)	(No time and group interaction measures, as interaction was not significant in model with interaction of EORTC QLQ-C30 scales: cognitive functioning, social functioning, nausea and vomiting, dyspnea, appetite loss, constipation, financial difficulties and for level of physical activity (FBACQ))
Leensen, 2017	After rehabilitation, and 6, 12 and 18 months after baseline (post-rehabilitation)	After rehabilitation (only measures of muscle strength and cardiorespiratory fitness) •VO2 peak (ml/min/kg) •1RM leg press (kg) •1RM deltoid pulley (kg) •Maximal short exercise capacity • (steep ramp test) (W) Differences between baseline and 6 months: •Rate of return to work RTW •Perceived importance of work •WLQ, time management •WLQ, physical demands •WLQ, production demands •MFI, general fatigue •MFI, physical fatigue •MFI, reduced motivation •MFI, reduced activity •MFI, total score •Physical activity EORTEC QLQ-C30: •Role functioning •Cognitive functioning •Fatigue •Nausea Differences between 6 and 18 months: •Rate of return to work RTW •Perceived importance of work •Work ability (first item of WAI) •Self efficacy regarding RTW •MFI, general fatigue •MFI, physical fatigue •MFI, reduced motivation •MFI, reduced activity •MFI, mental fatigue •MFI, total score •Physical activity EORTEC QLQ-C30: •Physical functioning •Role functioning •Social functioning •Fatigue •Global health	After rehabilitation (only measures of muscle strength and cardiorespiratory fitness) •Maximal workload (W) Differences between baseline and 6 months: •Work ability (first item of WAI) •Self efficacy regarding RTW •WLQ, mental-interpersonal demands •MFI, mental fatigue EORTEC QLQ-C30: •Physical functioning •Emotional functioning •Social functioning •Pain •Global health Differences between 6 and 18 months: •WLQ, time management •WLQ, physical demands •WLQ, mental-interpersonal demands •WLQ, production demands EORTEC QLQ-C30: •Cognitive functioning •Emotional functioning •Nausea •Pain
Seibaek, 2016	12 months after baseline (post-rehabilitation)	SF36: •Role physical •Vitality •Social dunctioning •Role emotional	SF36: •Physical functioning •Bodily pain •General health •Mental health •Sense of coherence
Thorsen, 2016	6 months after baseline (post-rehabilitation)	Patients who improved their work status at 6 months: EORTEC QLQ-C30: •QoL •Physical functioning •Role functioning •Emotional functioning •Cognitive functioning •Social functioning •Fatigue •Nausea and vomiting •Pain •Dyspnoea •Insomnia •Appetite loss Fatigue Questionnaire: •Physical fatigue •Mental fatigue •Total fatigue Patients who did not improve their work status at 6 months: EORTEC QLQ-C30: •QoL •Physical functioning •Role functioning •Emotional functioning •Fatigue •Appetite loss •Constipation •Diarrhoea •Financial difficulties Fatigue Questionnaire: •Physical fatigue •Total fatigue •Physical activity index	Patients who improved their work status at 6 months: EORTEC QLQ-C30: •Constipation •Diarrhoea •Financial difficulties Fatigue Questionnaire: •Physical activity index Patients who did not improve their work status at 6 months: EORTEC QLQ-C30: •Cognitive functioning •Social functioning •Nausea and vomiting •Pain •Dyspnoea •Insomnia Fatigue Questionnaire: •Mental fatigue

## Discussion

Our systematic review revealed positive effects of MOCR on the physical and/or psychosocial status of cancer patients. However, a variety of physical and psychosocial outcomes did not improve, or at least not significantly. Additionally, significant and non-significant rehabilitation effects were not consistent across studies. For example, several studies reported improvements in cardiovascular fitness, but the effects were observed through different metrics. Furthermore, there was insufficient evidence with respect to the long-term effects of MOCR on physical and/or mental health status of cancer patients as well as its effects on return to work status. Also, no evidence was observed that suggests that the effects of MOCR vary depending on the start of rehabilitation.

Our systematic review provides a comprehensive overview of the effects of MOCR on physical, psychosocial and return to work status. Similar results were obtained in one systematic review, but the review did not differentiate between inpatient and outpatient settings and focused only on multidimensional rehabilitation [13]. Another systematic review also observed that brief and focused multidimensional rehabilitation programmes are effective, but it also did not differentiate between inpatient and outpatient rehabilitation, and for several of the studies included, it was not clear how much time between the end of the primary active treatment (e.g. chemotherapy, radiotherapy or surgery) and the start of the rehabilitation had passed [14]. Therefore, with our search strategies focusing on MOCR, studies included in our review differed from those included in the previous reviews.

The absence of stratification by inpatient or outpatient settings in the two previously mentioned systematic reviews may be due to the fact that only few countries, in particular German-speaking countries (i.e. Germany, Austria and Switzerland), have a tradition of inpatient rehabilitation. However, as a result of political and structural changes, such as an aging population, an increasing number of cancer survivors and financial restrictions for inpatient care and outpatient rehabilitation become increasingly more important in these countries [28, 29]. One German epidemiological multicenter study with more than 4000 cancer patients from acute care hospitals, outpatient facilities and rehabilitation clinics compared QoL of patients with the general population. QoL was higher in the general population than in cancer patients, and QoL was higher in multidisciplinary rehabilitation and in an outpatient setting compared with the inpatient setting [30]. Besides for political and structural changes, MOCR meets the needs for improving QoL of each cancer patient considering the biopsychosocial model in understanding health. This model emphasizes that interconnections of biological, psychological and socio-environmental factors affect health, such that biopsychosocial care improves clinical outcomes, especially for chronic diseases [31]. Furthermore, rehabilitation that includes physical as well as psychosocial factors has been shown to be more effective on health, especially on pain management after cancer [32]. However, also intraindividual factors may play a role in rehabilitation outcomes [33].

As evidence is still limited, more studies are needed to strengthen the evidence of MOCR effects and to specify these effects on health to facilitate the development of evidence-based rehabilitation, national quality criteria and certified programmes. Nowadays, at least in Europe, rehabilitation is still not a well-established component of cancer control plans. The EUROCHIP-3 results showed that in 2011, 18 out of 25 European Union countries (72%) reported cancer rehabilitation in their national cancer plans [34]. This same study revealed that only four European Union countries had cancer rehabilitation guidelines in 2011 and two were preparing guidelines. A preliminary work of the present review consisted of a targeted internet search in English, German, French, Italian, Norwegian, Swedish, Netherlands, Estonian, Finish and Chinese with respect to national guidelines for outpatient cancer rehabilitation. It was observed that the situation is even worse; out of 15 countries that provided information, only six countries were identified as having a national guidelines for outpatient cancer rehabilitation (Netherlands [35, 36], Sweden [37], Denmark [38–40], Germany [41, 42], Austria [43] and UK [44]).

### Strengths and Limitations

The main strength of our review was the inclusion of both RCTs and before-after studies, which allowed for assessing a variety of outcomes, including return to work status, which was only assessed in before-after studies. However, only two before-after studies included a control group but had a high drop-out rate [23, 24]. Thus, improvements in physical and psychosocial status, as well as returning to work, could have occurred due to other reasons, e.g. passage of time, and not necessarily as a result of rehabilitation. Therefore, RCTs with higher methodological quality are needed to further evaluate the potential effects of cancer rehabilitation, especially in improving return to work status of cancer patients. A main limitation of the current systematic review was its narrative approach, which was not supplemented by meta-analyses. This was linked to the large heterogeneity and number of outcome measures, which made it difficult to compare the studies. Standards to assess rehabilitation effects and, hence, make assessments comparable across studies are urgently needed. Furthermore, the quality assessment showed that the overall methodology of the studies included was poor, partly due to the study design and partly due to methodological deficiencies of the studies.

Future randomized controlled trials should particularly analyze the long-term effects of MOCR and its effects in improving the return to work status of cancer patients. Due to the small number of studies with site-specific cancer, more research is needed regarding site-specific cancer rehabilitation programmes.

## Conclusion

The findings of the systematic review indicate that MOCR can potentially improve cancer patients’ physical and psychosocial status. But more research is needed, especially in regard to long-term outcomes, effectiveness on return to work status and on the associations depending on cancer type.
